# Upbeat nystagmus: a clinical and pathophysiological review

**DOI:** 10.3389/fneur.2025.1601434

**Published:** 2025-07-01

**Authors:** Vincenzo Marcelli, Beatrice Giannoni, Giampiero Volpe, Mario Faralli, Edoardo Marcelli, Michele Cavaliere, Anna Rita Fetoni, Vito Enrico Pettorossi

**Affiliations:** ^1^Department of Neuroscience, Reproductive Science and Dentistry, Section of Audiology, University of Naples “Federico II”, Naples, Italy; ^2^Department of Neuroscience, Psychology, Drug’s Area and Child’s Health, University of Florence, Florence, Tuscany, Italy; ^3^Department of Neurology, Ospedale San Luca di Vallo della Lucania, ASL Salerno, Salerno, Italy; ^4^Department of ENT, University of Perugia, Perugia, Italy; ^5^Faculty of Medicine and Surgery, University of Salerno, Salerno, Italy; ^6^Department of Medicine and Surgery, University of Perugia, Perugia, Italy

**Keywords:** UBN, oscillopsia, unsteadiness, vertigo, treatment

## Abstract

Finding a vertical nystagmus, especially when looking straight ahead, should alert the neurologist/neuro-otologist for other signs of cerebellar or brainstem dysfunction. Upbeat nystagmus (UBN) is a relatively uncommon neuro-otological finding that clinicians may encounter in patients presenting with vertigo. This phenomenon is closely linked to central vestibular dysfunction, making it essential for healthcare providers to recognize and interpret it promptly. Accurate identification of UBN can significantly aid in directing patients toward the appropriate diagnostic and therapeutic pathways. As our understanding of UBN’s pathophysiology has advanced, the clinical significance of this sign has become increasingly evident. It is now recognized that UBN can occur as an isolated finding or more frequently as part of a broader spectrum within defined clinical syndromes. This expanded knowledge has also opened the door to various therapeutic approaches tailored to the underlying cause. In our study, we want to provide as accurate a picture as possible about the origins and clinical presentations of UBN.

## 1 Introduction

First described by Stengel in 1935 (1), upbeat nystagmus (UBN) significantly impairs patients’ quality of life by producing disabling visual disturbances such as oscillopsia and gaze instability. These symptoms underscore the clinical importance of a thorough understanding of UBN and its management. However, UBN remains a relatively rare finding, limiting opportunities for clinicians and researchers to gain sufficient diagnostic and therapeutic experience. Moreover, UBN is frequently linked to lesions in key regions of the brainstem (particularly the medulla), as well as the flocculus and the brachium conjunctivum (BC). It often presents alongside with other neurological signs, such as saccadic contrapulsion or impaired smooth pursuit, further complicating its recognition and interpretation. Despite its clinical relevance, the neuroanatomical pathways responsible for upward vertical gaze remain incompletely understood. Much of the current knowledge about UBN derives from lesion-based and clinical observational studies (2), highlighting the need for an integrated synthesis of available evidence. Moreover, while individual publications address specific aspects of UBN, a comprehensive, unified resource is lacking, forcing clinicians to consult disparate sources and potentially leading to fragmented understanding. A consolidated review of UBN is therefore warranted to integrate current knowledge, elucidate underlying mechanisms, correlate clinical findings with neuroimaging data, and propose evidence-based approaches to diagnosis and treatment. Such a resource would be invaluable for clinicians, researchers, and patients alike, ultimately supporting improved care and advancing future investigations.

### 1.1 First descriptions of vertical nystagmus

Vertical nystagmus received limited attention in the early medical literature (3, 4) until 1921, when it was experimentally induced in animals by severing the anterior semicircular canal (SCC) and observed in clinical contexts such as profound bilateral visual loss, drug toxicity (e.g., barbiturates, quinine), multiple sclerosis, spasmus nutans, brainstem lesions (e.g., pontine and medullary tuberculomas and meningiomas), and encephalitis lethargica (5). While some authors emphasized the importance of distinguishing central from peripheral etiologies in cases of vertical or mixed nystagmus (6), Walsh (7) asserted that purely vertical nystagmus was a hallmark of central or cerebellar pathology. He described cases associated with Arnold-Chiari malformation, cerebellar ataxia, bilateral pyramidal tract signs, and hydrocephalus, reinforcing the central origin hypothesis.

## 2 Characteristics of UBN

Upbeat nystagmus is often a sign of the acute phase of a disease tending to resolve spontaneously before other ocular motor abnormalities. It is typically present in the primary gaze position, both in darkness and during fixation, and must be distinguished from nystagmus evoked exclusively by upward gaze secondary to a vertical gaze holding neural integrator lesions. The slow phases of UBN may have different waveforms, which may be linear, decaying due to a “leaky” neural integrator, or more commonly, increasing due to an unstable neural integrator (2). Unlike downbeat nystagmus (DBN), UBN is minimally affected by lateral gaze (8). In some cases, the upward fast phase may have a horizontal component alternately directed to the right or to the left, in which case it is configured as a “bow-tie nystagmus” (9) or have a torsional component. In this last case, it may be due to the unilateral BC lesion, which in health conveys excitatory upward-torsional eye movement signals from the anterior semicircular canals (10) or to an associated lesion of pathways controlling the VOR in roll (11).

### 2.1 Alexander law and UBN

Upbeat nystagmus (UBN) typically follows Alexander’s law, exhibiting maximal fast-phase amplitude in upward gaze. However, in some cases, it may paradoxically increase with downward gaze (12, 13) or even convert into downbeat nystagmus (DBN) during upward gaze (13). These atypical patterns have been associated with lesions of the interstitial nucleus of Cajal (INC), which makes unstable the vertical neural integrator responsible for gaze holding - an association supported by corresponding MRI findings (2).

### 2.2 Effect of convergence and vestibular stimulation on UBN

Convergence has a variable effect on UBN, being able to increase or decrease its intensity up to its suppression or in some cases transforming it into a DBN (14–17). At same time, vestibular stimulation, such as head-shaking and vibration, may reverse nystagmus direction. The changes in nystagmus patterns due to convergence and vestibular stimulation are likely due to disruptions in circuits that process linear acceleration signals from the otoliths, which are essential for generating the correct eye movements during head translation (18). A fundamental role in this is played by the translational vestibulo-ocular reflex (t-VOR), which during movement adjusts compensatory eye movements based on orbital position and vergence angle depending on the target position. In this way, during translation forward or backward the t-VOR generates horizontal movements for targets to the sides, vertical movements for targets above or below, and convergence/divergence for targets straight ahead. Key brain areas involved in these computations include the medial and inferior vestibular nuclei in the spinal cord, which mediate the velocity storage mechanism (19, 20), and their projections to the cerebellar nodulus, which computes the translational components of head movements (21). Disruption of these pathways or velocity storage mechanisms may explain the change in direction of nystagmus in response to convergence and vestibular stimuli, respectively (22).

### 2.3 Gravitational dependence of UBN

Changes in head position relative to space, and hence in macular input, can abolish (8, 23, 24) UBN, increase its slow-phase velocity (25) and change its direction, often transforming it into a DBN (26, 27). UBN often tends to disappear in the supine or prone position, where the effect of the gravitational vector on the maculae is different (8). In a patient with a focal hemorrhagic lesion of the left BC, anterior vermis and left anterior superior cerebellar hemisphere, UBN was also suppressed by a contralateral head tilt due to otolith-ocular reflex activation (23). In a patient affected with a lesion at the cervicomedullary junction due to multiple sclerosis, the spontaneous upward and counterclockwise nystagmus was suppressed by the prone position, tilting the head to either side while sitting, and turning the head to the right while in the supine position. Moreover, nystagmus direction was reversed by hanging the head straight and turning the head to the left while the patient was in the supine position (24). Change from UBN to DBN were observed in the prone position in a patient with a lesion of the BC and moving from upright to a head-hanging position a patient with cerebellar vermis atrophy (28).

### 2.4 Spontaneous transformation of UBN in other nystagmus type

Another very peculiar feature of UBN is its possible spontaneous transformation, in the course of the disease, into a hemi-seesaw (29, 30), horizontal, torsional and above all DBN (17, 22, 24, 28, 31, 32). A very interesting case of the above phenomenon is Wernicke’s encephalopathy. The initial thiamine deficiency damages the perihypoglossal nuclei, particularly the nucleus intercalatus of Staderini (SIN) and the nucleus of Roller (RN) more than the PMT nuclei, resulting in a downward slow-phase bias and UBN. Over time, recovery is more complete in the perihypoglossal than in the PMT nuclei, which fails to provide excitatory input to the cerebellar flocculus. The consequence is that the cerebellar flocculus no longer exerts inhibitory control over upward slow-phase pathways resulting in an upward slow-phase bias and hence in DBN (30). Obviously, also the contiguity of the areas responsible for the vestibular syndrome in the sagittal plane may also justify the change in nystagmus direction (33, 34). Overall, changes in nystagmus direction with convergence, head tilt, vestibular stimuli or simply over the course of the disease reflect the tightly interconnected network that maintains balance in the pitch plane.

### 2.5 Neuronal circuits responsible for upward and downward postural and ocular reflexes

The neuronal circuits responsible for upward and downward postural and ocular reflexes are distinct within the central nervous system. These specialized pathways underlie the generation of different types of vertical nystagmus. Specifically, damage to the circuit facilitating upward compensatory and downward anticompensatory eye movements can result in UBN, while lesions affecting the circuit that facilitates downward compensatory and upward anticompensatory responses can lead to downbeat nystagmus (DBN) (35).

The effectiveness of the circuits controlling upward and downward vestibulo-ocular reflexes is not equal. Actually, upward eye movement reflexes are stronger than downward eye movement. Support for this assertion is the observation that optokinetic nystagmus and optokinetic after nystagmus as well as nystagmus induced by vertical rotation (36, 37) predominate for upward direction. Further confirmation is provided by the fact that the time constant for DBN is about 15 s, which is like that of horizontal nystagmus, whereas for UBN nystagmus it is about 8 s.

**Figure 1:**
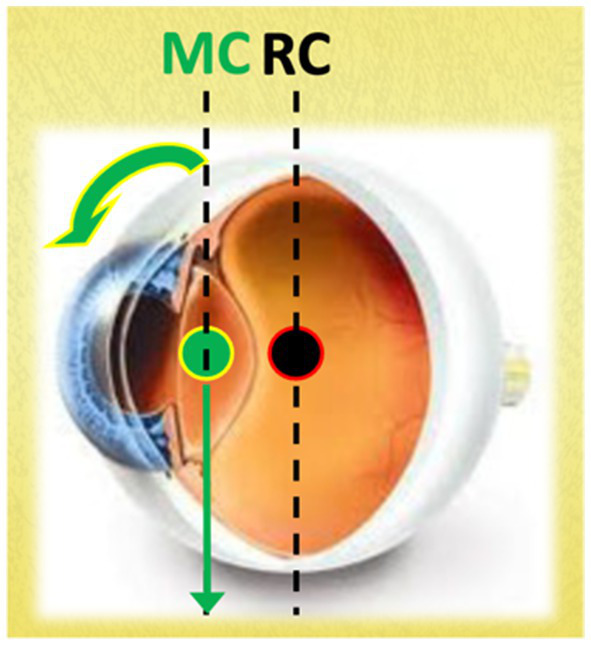
The figure illustrates that the vertical line passing through the center of mass (MC) is positioned anterior to the vertical line passing through the center of rotation (RC). In this configuration, with the subject in an upright position and the head aligned with the trunk, gravity counteracts the upward vertical VOR while facilitating the downward vertical VOR. This asymmetry must be counterbalanced by specific neural pathways.

One reason for this vertical reflex asymmetry may be due to the anatomy of the eye. In fact, at least in the cat, the center of mass of the eyeballs is located anterior to the center of rotation (38, 39) and therefore, with subject in an upright position and the head in line with the trunk, the force of gravity limits the vertical VOR upwards and favors the vertical VOR downwards, creating a clear and maximum imbalance between the two vertical eye movement systems (Figure 1). On the other hand, the load and viscosity of the eye alone may not necessitate such a pronounced upward reflex preponderance.

An alternative explanation considers that gaze stability is maintained not only by the vestibulo-ocular reflex (VOR) but also by the vestibulocollic and cervicocollic reflexes (40). In the case of head reflexes, the influence of gravity is significantly greater than that acting on the eyes, requiring a robust upward enhancement. Consequently, the observed upward bias in the ocular reflex may also serve to compensate for potential gain and phase deficits in the head reflex, thereby providing effective counteraction to gravitational forces.

In addition, the upward preponderance should be modulated depending on the position of the head re-gravity, so that an influence from the otolithic signals, sensing the orientation of the gravity vector, is required for inhibiting or enhancing the upward responses depending either on the direction of the gravity vector and on the load and viscosity in the motor system involved.

### 2.6 Peripheral source of asymmetry in the vertical eye movement reflexes

At the peripheral level, the upward preponderance in vertical eye movement reflexes is supported by the anatomical and physiological properties of the vertical semicircular canals (VSCCs) (41). VSCC activation occurs via an ampullofugal stimulus, with the receptors of the anterior SCCs being more effectively stimulated by downward rotations and the receptors of the posterior SCC (PSCCs) responding more strongly to upward rotations. Because the anterior SCCs (ASCCs) are more closely aligned with the sagittal plane than the posterior canals, the signals from vertical head rotations are inherently asymmetric, producing a stronger response during downward rotations.

### 2.7 Central source of asymmetry in the vertical eye movement reflexes

The most important central pathway facilitating upward eye movements is the crossing ventral tegmental tract (CVTT). Additional areas potentially involved in such an asymmetry include a circuit consisting of Superior Vestibular Nucleus (SVN), the RN and the SIN, and a circuit in which the PMT are involved.

## 3 Pathophysiology for UBN

A damage to one of these circuits would reveal the underlying downward imbalance leading to UBN.

### 3.1 The CVTT

This complex crossing pathway, responsible for upward eye movement facilitation, originates in the upper pole of the SVN, courses predominantly in the pons, then in lower midbrain tegmentum and finally reaches the III (oculomotor) nucleus (42, 43).

Afferences to SVN come from the anterior semicircular canals, both directly both indirectly through the flocculus, via the interstitial nucleus of the vestibular nerve (2); the maculae, both directly and through the flocculus; the caudal medulla, in which RN and SIN ‘s receives a collateral pathway from the CVTT and projects to the flocculus, for plausible feedback control; the flocculus, which receives inputs from the caudal medulla, ASCCs, maculae and visual apparatus and projects to the SVN, ensuring control of the circuit (44).

**Figure 2:**
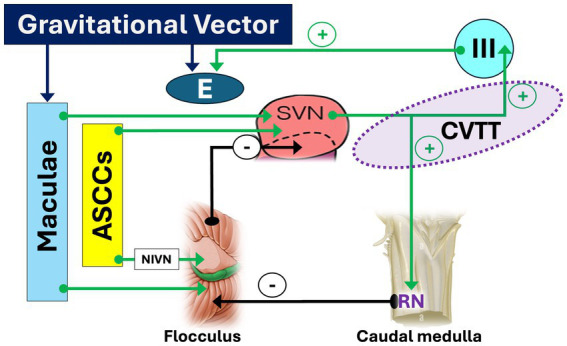
Pathways and circuits facilitating upward slow phase. Afferent inputs to the superior vestibular nucleus (SVN) originate from the anterior semicircular canals - both directly and indirectly via the flocculus through the interstitial nucleus of the vestibular nerve (NIVN)–as well as from the maculae, following similar direct and indirect pathways via the flocculus. From the upper pole of the SVN, the crossing ventral tegmental tract (CVTT) emerges and projects to the oculomotor (III) nucleus. Additionally, a collateral branch of the CVTT reaches the nucleus of Roller (RN) in the caudal medulla, which, by inhibiting the flocculus (−), modulates its inhibitory influence on the SVN. *The “+” sign indicates facilitatory effect of CVTT for upward slow phase*. LVN: lateral vestibular nucleus.

The CVTT can be regarded as a true anti-gravitational pathway controlled by macular input (Figure 2) which constantly modulate the tonic activity of the oculomotor nucleus based on the orientation of the head and of the gravitational vector, counterbalancing gravitational forces in relation to the head and eye’s static position and correcting the eyeball’s vertical inertial asymmetry. When the head is in upright position, the gravitational vector facilitates the CVTT so that the upward eye responses are potentiated. In prone or supine positions, the macular input changes and diminishes its facilitatory effect in the CVTT, so that the eye positions balance is therefore guaranteed only by the activity of the pathways that run in the MLF (see below). Finally, in the vertical head-down position, the upward facilitatory effect of CVTT will be minimal or absent and the gravitational vector will tend to favor upward eye movement relative to the head. In absence of CVTT-like circuitry compensating for such an eye movement due to gravity, an upward slow phase and chin-beating nystagmus may occur (45–47). Moreover, by providing the eye elevator muscle motoneurons with precise tonic activity, the CVTT integrates the function of the excitatory MVN-MLF pathway which, in the absence of gravity, would theoretically be sufficient by itself to manage the vertical movements of the eye. To put it more clearly: the excitatory MVN-MLF pathway controls upward eye movements, calculating the eye velocity relative to the orbit in response to rotational and/or translational head movements. The CVTT adjusts these parameters based on the instantaneous gravitational vector, i.e., the spatial position of the head.

The existence of a neuronal circuit facilitating upwards eye movements has been confirmed by numerous experiments performed in hypogravity and hypergravity (48–50) and above all from the clinical findings.

### 3.2 The SVN-RN/SIN-flocculus circuit

The SVN is also controlled by a loop located within the dorsal caudal medulla, consisting mainly of the RN and the SIN, both belonging to the perihypoglossal nuclei (PHN), and the flocculus. For completeness, it is worth mentioning the nucleus prepositus hypoglossi (NPH), which is also part of the PHN but does not participate in this specific circuit.

- Sublingual nucleus of Roller (RN). This very small nucleus is particularly well developed in higher primates such as chimpanzees and humans (51). It is located just ventral to the cranial tip of the hypoglossal nucleus and appears to be the better candidate to play a role in upward eye movements (10). In health subject, it receives strong excitatory projections from the SVN (52) and send inhibitory projections (51) to the flocculus (53) which in turn finally sends back inhibitory projections to SVN (53–55). A medullary lesion affecting the RN disrupts this inhibitory control over the flocculus, leading to its disinhibition. Consequently, this determines a strong inhibition of the SVN, impaired processing of input from the anterior semicircular canals (ASCCs), loss of tonic activity in the superior rectus and inferior oblique muscles, a downward drift of the eyes, and the emergence UBN (53, 55).
- Nucleus intercalatus of Staderini (SIN). This nucleus is located between the hypoglossal nucleus and dorsal nucleus of the vagus nerve (56) and creates a circuit that overlaps with the one described for the RN (57). The strong connections existing among perihypoglossal nuclei and flocculus, paraflocculus and nodulus (58, 59) suggest their role as the vertical cerebello-vestibular integrator involved in vertical gaze holding (53, 60–62). Upbeat nystagmus due to SIN involvement has been demonstrated in unilateral medial medullary infarction (60, 61), chronic lateral medullary infarction (63) multiple sclerosis (57), and cavernoma in the medulla oblongata (64). Despite the data presented, we believe it is right to remember that some authors do not fully agree with the gaze holding function; in fact, a patient with a dorsal paramedian lesion involving the SIN showed a constant-velocity slow phase rather than exponential decay, suggesting that this was not a nystagmus due to vertical gaze holding deficit (65).

### 3.3 The “cell groups of the paramedian tracts” (PMT)

PMT is a collective term used to refer to clusters of neurons scattered along the midline fibers tracts in the pons and medulla (66, 67) which receives inputs from all known premotor cell groups of the oculomotor system (52, 68–73) and project to the flocculus, paraflocculus, and vermis of the cerebellum (50, 66–68). The PMT contributes to neural integrator function by relaying eye movement signals to the vestibulocerebellum (67, 74). Particularly, the mid-medullary PMT-cell group in the monkey, the nucleus pararaphales (75, 76) receives vertical eye position signals from the interstitial nucleus of Cajal and therefore relays vertical eye position signals to the floccular complex. Thus, medullary lesions that affect the nucleus pararaphales may result in UBN (33, 77–81) and vertical gaze-evoked nystagmus (GEN). In addition, the PMT provide the cerebellum with a form of efference copy (or corollary discharge) of eye movement commands for the optimization of gaze control, including the fidelity of the neural integrator for eye movements or more long-term adaptive control of eye movements (74, 82).

### 3.4 The brachium conjunctivum

For the sake of completeness, we also report the crossing path of the BC, which has long been considered responsible for UBN. This pathway arises from the SVN, runs rostrally in the caudal tegmentum, and decussate in the caudal midbrain before projecting projects to the III (oculomotor) nucleus, which excites the motoneurons of the superior rectus and inferior oblique muscles, bilaterally. For a long time, this pathway was thought to be involved in the excitatory control of upward eye movements in the rabbit (83), until it was shown that this pathway was most likely confused with the CVTT, since these tracts are very close to each other in the lower pontine tegmentum (38, 39), and this is valid also in humans (23, 84, 85). Clinical data would confirm that a lesion of the BC does not determine an up-beat nystagmus consequent to the imbalance due to the predominance of the tonic activity of the system controlling downward movements as one would expect (86) but rather a down beat GEN (87) or a positional downbeat nystagmus (88). Moreover, some cases of spontaneous UBN due to involvement of the region through which the BC passes are characterized by large median tumoral or hemorrhagic lesions, always associated with damage to the cerebellar vermis, which in itself may lead to UBN (2, 78).

### 3.5 What is the role of the pathways that run through the medial longitudinal fasciculus?

To better understand the role of the pathway running through the medial longitudinal fasciculus (MLF) in the genesis of UBN, valuable insights can be gained from patients with lesions of this pathway, in whom high-acceleration head rotations reveal an asymmetrical vertical vestibulo-ocular reflex (VOR) gain deficit. Specifically, upward VOR gain is less severely affected than downward VOR gain (89, 90). This finding supports the notion that upward VOR signals are transmitted not only via the MLF but also through extra-MLF pathways (91).

Additional noteworthy observations in such patients include the absence of spontaneous vertical nystagmus and the presence of predominantly upbeat GEN (53, 89–91). The lack of spontaneous vertical nystagmus suggests a relatively symmetrical role of the MLF in vertical VOR control. Meanwhile, the presence of upbeat GEN confirms MLF damage, indicating that the lesion deprives the interstitial nucleus of Cajal—a key component of the vertical neural integrator—of vestibular input integrator (2).

Finally, the induction of UBN following an MLF lesion requires selective or predominant damage to the bilateral pathways responsible for the upward VOR, while sparing those involved in the downward VOR. This selective damage may explain why upbeat nystagmus is infrequently observed in cases of internuclear ophthalmoplegia (24).

## 4 Symptoms of upbeat nystagmus syndrome

The symptomatology of Upbeat Nystagmus Syndrome (UBNS) is essentially characterized by oscillopsia, motion illusion and sagittal imbalance. Oscillopsia, the most disabling symptom, is caused by the displacement of images on the retina due to the involuntary slow phase of nystagmus. In the absence of an efferent copy, which is generated only in the presence of a voluntary movement, the pathological displacement of the eyes creates a motion illusion of the visual scene in the vertical plane. The illusion is less intense compared to the magnitude of the slow-phase eye movements (92) presumably due to a reduction in the sensitivity of visual motion perception which is useful to mitigate the annoyance of illusory oscillations. Consequence of motion illusion due to oscillopsia is a compensatory vestibulo-spinal reflex (VSR) that contributes to the sagittal imbalance. By analogy with other vestibular vertigo syndromes, one would expect a tendency to fall forward due to motor compensation of an apparent backward tilt. In fact, under physiological conditions, extension of the head and/or trunk activates the vertical VOR, which is responsible for a slow downward phase and then the vestibulo-collic and vestibulo-spinal reflexes to compensate for the extension. Under pathological conditions, the tonic asymmetry generated not by the subject’s movement but by the central vestibular pathways dysfunction causes a downward eye movement and generates a retropulsion misperception characterized by an illusory head and/or body extension. The resulting postural compensation aims to compensate for the illusory extension, which is characterized by retropulsion and postural oscillations in the posterior–anterior direction. Contrary to these physiopathological premises some patients behaved differently, showing a tendency to fall backwards, in the same direction as patients with downbeat nystagmus (11).

## 5 Additional oto-neurological signs

Due to the presence of spontaneous upbeat nystagmus, smooth pursuit in the vertical plane is altered, constituting a “false” (extrinsic) alteration. On the other hand, the alteration of smooth pursuit is greater than would be expected from spontaneous nystagmus alone and the specific impairment of downward vertical pursuit suggests a direct and more extensive lesion affecting the pathways involved (93). Based on the possible associated lesions, it is possible to highlight other possible signs. For example, in patients with Wernicke encephalopathy a bilateral and symmetric loss of the horizontal VOR and a horizontal GEN can be highlighted due to the involvement of the NPH/MVN complex, which is located medial to the medulla and just below the area postrema (30, 94). Another possible finding associated with upbeat nystagmus is saccadic contrapulsion, characterized by hypermetria (overshooting) away from the side of the lesion and hypometria (undershooting) toward it. This phenomenon may result from the disruption of either: (1) the climbing fiber tracts that originate in the inferior olivary nucleus and project to the cerebellar vermis before crossing the midline (77); (2) the outputs from the fastigial nucleus that pass through the BC (85, 95).

## 6 Sites of lesions

**Figure 3:**
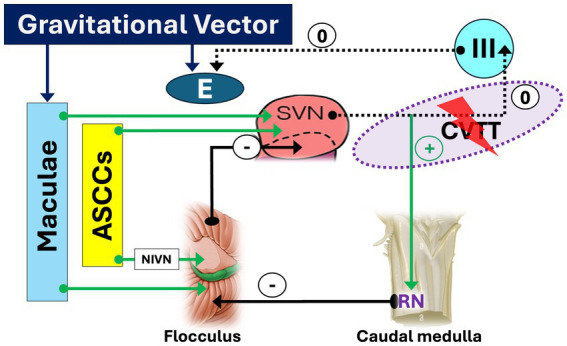
Crossing ventral tegmental tract (CVTT) lesion facilitating down slow phase and upbeat nystagmus. A lesion affecting the segment of the CVTT directed to the oculomotor nucleus deprives the elevator muscles of inputs from the anterior semicircular canals (ASSCs). As a result, a downward-directed slow phase and an upbeat nystagmus (UBN) will occur. *The sign “0” indicates the disappearance of the facilitatory effect of CVTT for upward slow phase*.

**Figure 4:**
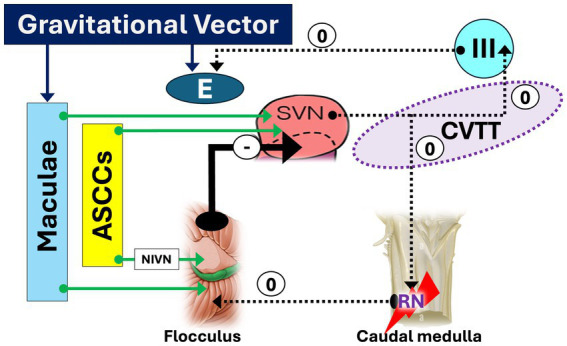
Caudal medulla lesion facilitating down slow phase and upbeat nystagmus. A medullary lesion affecting the nucleus of Roller (RN) disrupts its inhibitory control over the flocculus, resulting in disinhibition. Consequently, this leads to strong inhibition (heavy arrow) of the superior vestibular nucleus (SVN), impaired processing of input from the anterior semicircular canals (ASCCs), loss of tonic activity in the superior rectus and inferior oblique muscles, a downward drift of the eyes, and the emergence of upbeat nystagmus (UBN). *The sign “0” indicates the disappearance of the facilitatory effect of CVTT for upward slow phase*.

The sites of lesions responsible of upbeat nystagmus must be sought in:

1. Lesion of CVTT, which carries signals from the ASCCs to the elevator muscles of the eyes (Figure 3). Several possible sites of CVTT lesion have been demonstrated in humans:
  - Lesions affecting the upper pole of the SVN and the initial portion of the pathway, in the posterolateral region of the inferior pontine tegmentum (96)
  - Lesions affecting the intermediate segment of the CVTT, near the decussation, in the central part of the pons (44)
  - Lesions affecting the final portion of the CVTT, in the anterior midbrain tegmentum (97)
  - Bilateral lesions affecting the anterior pontine tegmentum and the adjacent posterior basis pontis in the middle and upper pons (8, 98, 99).
2. Lesions affecting caudal medulla (77):
  - Lesion affecting the RN, which play a fundamental role in upward eye movements (33) (Figure 4)
  - Disruption of the mid-medullary nucleus pararaphales of the PMT, involved in vertical gaze holding (67, 74)
  - Lesions affecting the SIN, also involved in vertical gaze holding (53, 60–62) whose role is however questioned (65).
3. Rarer sites of lesions are the anterior vermis of the cerebellum (2, 24, 28, 78) and the thalamus (24), moreover without a precise explanation of the mechanisms.

## 7 Etiology

The most frequent central causes of UBNS are reported in Table 1.

**Table 1:** Most frequent causes of UBNS.

*Vascular pathologies* Infarction of medulla (27, 60, 61, 64, 77, 111–118) Infarction of pontomesencephalic junction (10, 119) Infarction of BC (84, 85) *Inflammatory and autoimmune diseases* Meningitis Brainstem encephalitis (120) Multiple sclerosis Anti-GAD antibodies, including stiff-person syndrome (121–123) Middle ear disease (124) Variants of Guillain-Barre syndrome such as Bickerstaff encephalitis and Miller-Fisher syndrome (121, 125, 126) *Paraneoplastic* Anti-Hu antibodies due to pancreatic endocrine neoplasm (26) Anti-Ma2 antibodies encephalitis (127) *Neoplasm* Tumor of cerebellar vermis (128) Tumor of the medulla (8, 129) *Hereditary neurodegenerative disease* Episodic ataxia type 2 (positionally induced) Ataxia telangiectasia Cerebellar degeneration (130) Lodder-Merla syndrome type 1 (131)	*Toxic conditions* Wernicke’s encephalopathy (13, 32, 132, 133) Tobacco and nicotine intoxication (46, 134, 135) Organophosphate poisoning (136) Organoarsenic poisoning (137) *Visual pathway diseases* Leber’s congenital amaurosis (2, 138) Congenital disorders of the anterior visual pathways (139) *Malformative conditions* Arnold Chiari disease (140) *Miscellanea* Central diabetes insipidus (141) Pelizaeus-Merzbacher disease (142) Fisher’s syndrome (143) Transient finding in infants (144, 145) Congenital upbeat nystagmus (146–148) Creutzfeldt-Jacob disease (149) Amitriptyline discontinuation (150) Hydrocephalus (24) Hyperemesis gravidarum (151–153)

### 7.1 UBN in peripheral vestibular diseases

Although UBN is typically associated with central vestibular or brainstem lesions, rare cases of peripheral origin have been documented. Ichimura and Itani (100) reported a case of persistent positional UBN in a patient with bilateral posterior canal benign paroxysmal positional vertigo (BPPV) due to canalolithiasis. The nystagmus was elicited during the transition from an upright seated to a straight supine position, characterized by a latency of approximately 2 s and a maximum duration of 110 s. The absence of neurological signs, normal brain imaging, and spontaneous resolution supported a peripheral etiology. Similar cases have been described in earlier literature (101, 102). More frequently, a mixed spontaneous nystagmus with an upbeat component is seen in peripheral vestibular dysfunction. For instance, Fetter and Dichgans documented such a case in superior vestibular neuritis (VN) (103–105). In a retrospective study by Ling et al. (106), 43 patients with UBN were reviewed, and peripheral vestibular disorders were identified in 14 (32.6%) of them. These included 10 cases of superior acute unilateral vestibulopathy (AUVP), 1 complete AUVP, 1 probable labyrinthine infarction, 1 isolated acute unilateral utricular vestibulopathy, and 1 probable Ménière’s disease. Therefore, while rare, peripheral causes of UBN must be considered. A comprehensive diagnostic approach - integrating clinical history, symptomatology, neurological and neuroimaging evaluation, and especially what we call “semeiological features with different diagnostic weight” (e.g., nystagmus direction and characteristics, smooth pursuit, saccadic eye movements, skew deviation) are essential for accurate localization and differentiation.

Table 2 *reports key points for distinguish between central or peripheral origins of the UBN.*

**Table 2:** Key points of peripheral and central UBN.

UBN	Peripheral	Central
Spontaneous	Very rare	Frequent
Positional	Frequent	Frequent
Presence in primary eye position	Yes	Yes
Presence of horizontal components	Rare	Possible (bow-tie nystagmus)
Presence of torsional components	Frequent	Possible
Modification by lateral gaze	Frequent, for torsional component	Rare
Alexander law	Yes	Variable
Slow phase velocity trend	Linear	Linear, increasing or decreasing
Visual fixation inhibition	Yes, for vertical component	No
Modification by convergence	No	Yes
Modification with different head positions	Only in BPPV	Often
Association with other oculomotor abnormalities	No	Often

## 8 Treatment of UBN and related symptoms

Management UBN may be either causal or symptomatic, depending on the underlying etiology. In cases of toxic, metabolic, deficiency-related, or autoimmune conditions, identification and elimination of the triggering factor may allow for resolution or reduction of the nystagmus. However, when causal treatment is not feasible and spontaneous remission does not occur, symptomatic therapy becomes necessary.

The primary aim of symptomatic treatment is to improve visual stability and reduce oscillopsia, while preserving normal ocular motor function, and enhancing postural control.

Although carbamazepine, an antiepileptic agent, has occasionally been reported to reduce UBN (107), the most commonly employed pharmacological agents include baclofen, 4-aminopyridine (4-AP), and memantine, which may be used individually or, in cases of insufficient efficacy, in various combinations.

Baclofen, a GABA B receptor agonist, reduces the slow-phase velocity of nystagmus and alleviates oscillopsia (108) by potentiating the inhibitory influence of the vestibulocerebellum on vestibular nuclei.

4-aminopyridine (4-AP), a potassium channel blocker, has demonstrated efficacy in attenuating UBN and associated visual disturbances, restore impaired upward smooth pursuit (93, 109), and modulate macular-driven vertical gaze control (27). The lack of efficacy in darkness suggests its action may involve facilitation of visually dependent parallel pathways that suppress UBN in lighted conditions. Moreover, 4-AP may increase cerebellar Purkinje cell excitability, thereby enhancing parallel compensatory circuits.

Memantine, a non-competitive NMDA receptor antagonist, may be beneficial in selected patients (110).

In summary, pharmacological management of UBN should be tailored to the etiology and symptom burden, with consideration given to both targets and patient-specific responses.

## 9 Conclusion

A highly significant finding, often indicative of central vestibular dysfunction, is UBN. Only recently has its diagnostic importance been widely recognized, thanks to advancements in research on its origin and pathophysiology. We now have a detailed understanding of the neural structures responsible for the precise control of eye movements in the vertical plane, allowing us to focus on these structures when encountering UBN.

Today, UBN is considered a highly localizing pathological sign, and its identification should prompt thorough neuroimaging studies to detect the most common acute or chronic structural causes.

As a result, therapy for UBN and related symptoms can be causal. When this is not feasible, treatment can be symptomatic.

The goal of our study is to provide a comprehensive and detailed overview of UBN and to offer possible explanations for the different functional aspects observed in the presence of this finding. This paper would serve as a valuable resource not only for specialists evaluating these patients in a clinical setting but also for general practitioners who may encounter this sign and pathology in non-specialized contexts, such as in emergency care.
